# The beauty and the morbid: fungi as source of inspiration in contemporary art

**DOI:** 10.1186/s40694-016-0028-4

**Published:** 2016-11-29

**Authors:** Corrado Nai, Vera Meyer

**Affiliations:** 1 Department Applied and Molecular Microbiology, Institute of Biotechnology, Berlin University of Technology, Gustav-Meyer-Allee 25, 13355 Berlin, Germany; 2 Federation of the European Microbiological Societies (FEMS), Delftechpark 37a, 2628 XJ Delft, The Netherlands

**Keywords:** Science and art, Bioart, Contemporary art, Fungi, Fungal-based materials, Burial suit

## Abstract

The arts have the power to irritate, to provoke and to let us think and dream about the impossible. The relationship of the arts and fungi is not immediate; however, fungi are ideal subjects for artists. They are both visible and invisible. They irritate. They evoke within each of us different feelings and inner pictures. Some are perceived as disgusting or dangerous because associated with dirt or death. Others are appreciated for their unique and delicious taste in our eating culture. Microbiologists further consider them as useful for industrial exploitation or per se as interesting because they are gratifying objects to study basic phenomena of life. To stimulate a fertile and interdisciplinary dialogue between artists and fungal scientists, we here present some examples of the inspirational powers of fungi and fungal science for contemporary art. Astonishing, poetic and perplexing artistic works could release scientific creativity and overcome the boundaries between art and science.

“Ever tried. Ever failed. No matter. Try Again. Fail again. Fail better.” Samuel Beckett (1906–1989) describes in his 1983 novella *Worstward Ho* [1] the very essence of the artistic process itself. Every person who has ever worked in a research environment can relate to those words too. Negative results, hard-to-interpret data and technical troubleshooting are a conspicuous part of the scientific process of every experimenter, regardless of her/his experience or the nature of the scientific question. And as such they are also the necessary prelude to the satisfaction of a successful experiment and to the elation of discovering something no one has ever seen before. Failures are part of the joys of experimenting. To negate this is to deny the functioning of science itself. By the words of Jules Verne (1828–1905): “Science, my boy, is made up of mistakes, but they are mistakes which it is useful to make, because they lead little by little to the truth” [2]. And to say it with the words of Albert Camus (1913–1960): “La lutte elle-même vers les sommets suffit à remplir un cœur d’homme. Il faut imaginer Sisyphe heureux” [3]—“The fight itself towards the summits suffices to fill a heart of man. We have to imagine Sisyphus as a happy man” (variant translation).

## The blurred boundary of science and art

There used to be a time when science and art went hand in hand. Perhaps because “Man cannot do without beauty” [4] or perhaps because both essentially have the same aim: to investigate and understand nature and (as part of it) ourselves. Still, insights into the world we are living in are explored by different means and generate different knowledge and experiences: rational, materialistic ones (science) and emotional, vivid ones (art).

In the Renaissance period, Leonardo da Vinci (1452–1519) combined the arts and science as a painter, inventor, sculptor and mathematician. His artistic works were the result of his scientific investigations. Much later, Alexander von Humboldt (1769–1859) documented his voyages as explorer and naturalist with beautifully accurate (or accurately beautiful) drawings. Many more examples can be made of thinkers who contributed significantly in the past to the advancement of both science and the arts. Several reasons could be ascribed to that. Curricula where more encompassing and scholars were trained also in classical disciplines, something which is nowadays not possible anymore due to the high specialisation and thus fragmentation of scientific and artistic disciplines. Intellectuals belonged to elitist circles and personal connections among representatives of different fields were nurtured, as for the friendship between the painter Johannes Vermeer (1632–1675) and the “father of microbiology” Antonie van Leeuwenhoek (1632–1723), contemporaries of the Golden Age of the Baroque, the period following the Renaissance. During these days, scientific insights into nature were limited and all knowledge could be conveyed through curricula. As exchange between scientists and artists was very intense and lively, many intellectuals were generalists.

These educational, professional and social milieus are today radically different. Scientists became specialists with very limited knowledge of the other’s disciplines, tools and technologies. C.P. Snow (1905–1980) argues in “*The two cultures and the scientific revolution*” [5] that the intellectual life of the modern Western world became separated into two cultures—science and the humanities—and that this separation hinders us to solve problems of mankind. Indeed, open-minded personalities of scientists and much translation work is nowadays necessary to run research projects involving multidisciplinary, interdisciplinary and transdisciplinary approaches which are key to understand complex phenomena. And the proactive exchange between science and art? Almost gone. This discrepancy shines through the words of Max Planck (1858–1947): “Experiment is the only means of knowledge at our disposal. Everything else is poetry, imagination.” But is he also encompassing artistic experimentations? And where does the creativity drive for innovative experiments come from, if not from the power of imagination?

In the preface to his autobiographical book *LSD*—*My Problem Child* [6], Albert Hofmann (1906–2008) describes how in his boyhood he occasionally perceived nature with intense, almost mystical sensations. Reflecting on his impossibility in communicating these experiences by artistic means due to his lack of vocational inclinations toward painting or poetry, he often anticipated his very personal failure. Recalling his career, he considered a lucky accident that his vocational interests were directed towards (fungal) natural products and that he discovered LSD and its psychoactive effects, able to open wide the doors of perception.

Luckily, many artists and artistic fields get nowadays creative impulses and become technologically empowered by scientific breakthroughs to investigate nature using artistic methodologies. Artistic fields that became inspired by science are as various as photography (e.g. in the nature-inspired captures by Rosamund Purcell, documented in the movies *An Art that Nature Makes*), music (e.g. in singer-songwriter John K. Samson’s *When I Write my Master’s Thesis* or in his more recent *Postdoc Blues*; or also in the transliteration of the DNA sequence or methylation patterns into music [7]), dance (as in the Dance Your PhD contest sponsored by the magazine *Science*), theatre (e.g. in the program fostered by the Alfred P. Sloan Foundation to exhort leading artists to explore scientific themes), the visual arts (e.g. in Salvador Dalì’s rendering of the DNA double helix in his *Butterfly Landscape*—*The Great Masturbator in a Surrealist Landscape with DNA*), illustration (e.g. in a Kickstarter campaign to produce a children book to show the contribution of woman in science throughout history), architecture (for example in the investigation of fungi as construction material), poetry (which, like science, gets at universal insights through metaphors and abstract thinking, or as testified by so-called “science slams”, an offshoot of “poetry slams”), literature (as for the “science-in-fiction” genre pioneered by the celebrated chemist Carl Djerassi), and the cinematic arts (as shown in the yearly festival by the non-profit organisation Imagine Science Film). The *Scenes* collection from Imagine Science Film for example gathers footage from scientists to visualize the beauty of scientific raw data images, with minimal interventions of post-production editing and human narrative.

## Fungi in the art scene

**Fig. 1:**
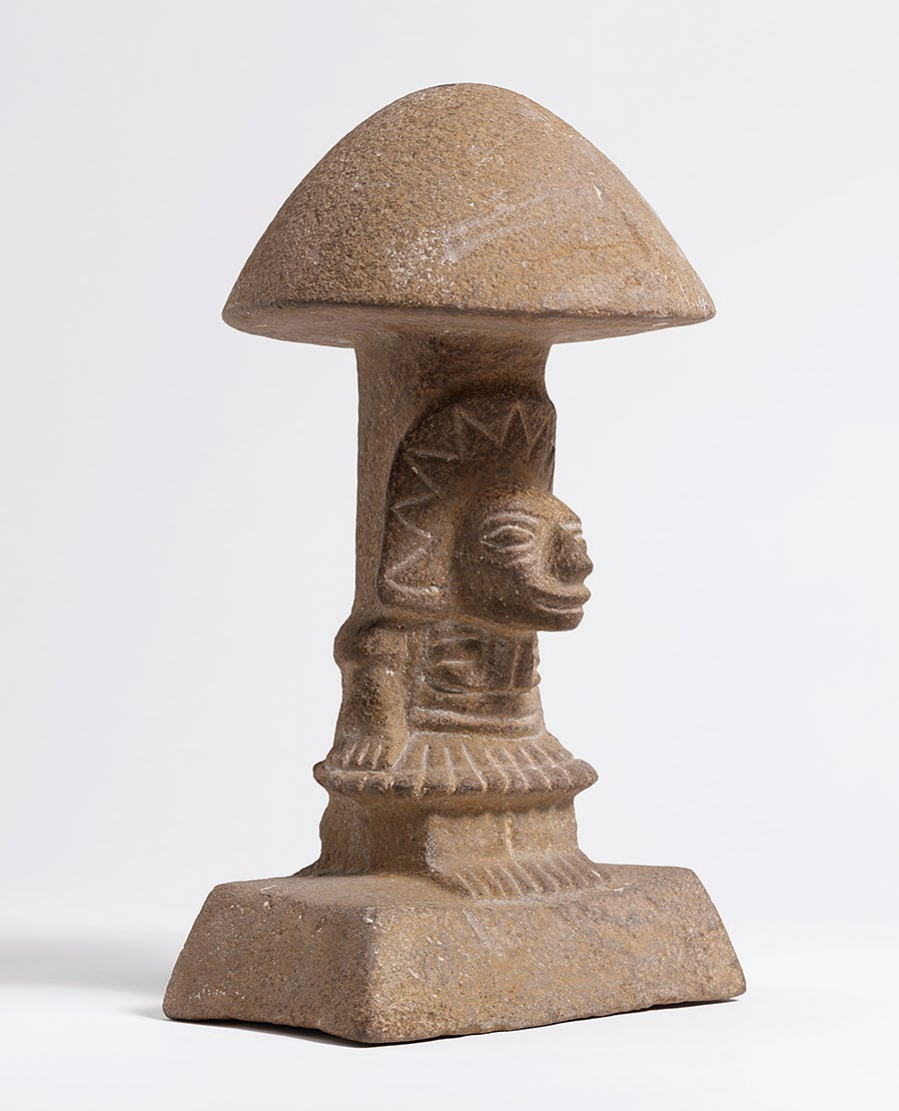
“Pilzstein” (mushroom stone) from El Salvador, 300 BC-250 AD. The carved figure is emblematic for the consumption of psychedelic mushrooms and a testimony of how psychoactive substances played a decisive part in the cultural history of Meso and South America. © Rainer Wolfsberger, Museum Rietberg Zürich

The relationship of fungi and the arts is not immediate. However, with their protean and versatile character, their morbid beauty and Janus-faced head, they recently became hot protagonists in the scene. One might think about the (ab)use of them—or of chemical derivatives thereof—as psychoactive substances as in the drug-fuelled counterculture and artistic hippy movement of the 1960s (“*you’ve just had some kind of mushroom/and your mind is moving low”* sang the Jefferson Airplane in *White Rabbit*). Interestingly enough, it has been proposed that the consumption of hallucinogenic substances of fungal origin might have contributed to the advancement of knowledge throughout history [6] long before the recreational use of fungal-derived drugs like LSD in the 1960s (Fig. 1). A recent example is given by the 1993 Chemistry Nobel Prize holder Kary Mullis who claimed that his consumption of LSD played a role in the discovery of PCR [8].

**Fig. 2:**
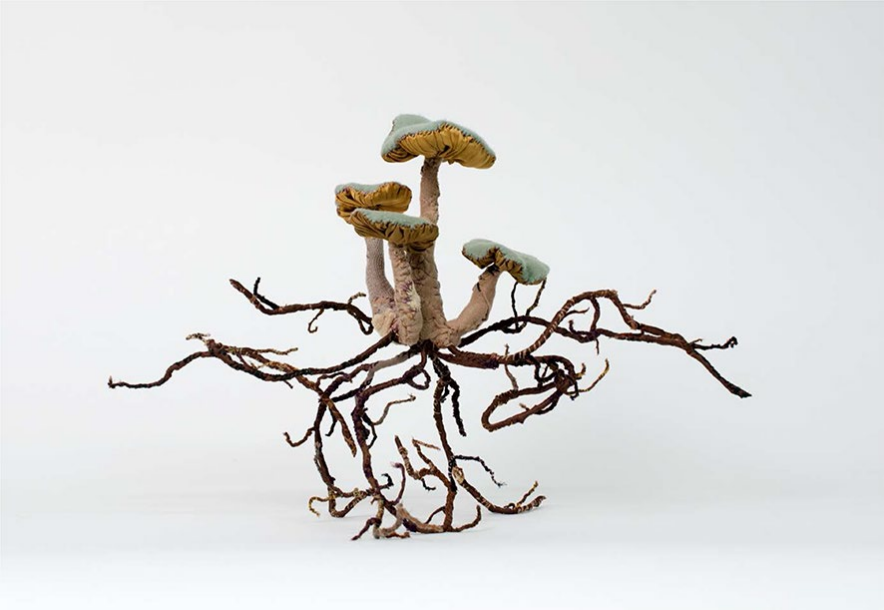
Anne Carnein, “without title”, 2015. Textile, yarn, wire. 20 × 30 × 25 cm. © Anne Carnein (courtesy of the artist)

Back into the art scene, the celebrated contemporary German artists Anselm Kiefer puts giant mushrooms at centre stage in his installation *Über Deutschland*, shown during his 2016 retrospective at the Pompidou Centre in Paris. There, the fungi are used as symbol of decadence, destruction, rebirth, and as source of inspiration for philosophers. The surreal and poetic sculptures of Anne Carnein presented at the 2016 Berlin Art Week—fungi, trees, flowers reproduced from pieces of clothing from the artist herself—let us think about the continuous cycle of becoming and dying, about moments full of deception and illusion (Fig. 2). In her *Objects not static and silent but alive and talking*, the artist Sonja Bäumel portrays the growth of mushrooms to provoke us to think about the static character of the objects surrounding us, as opposed to our own dynamic way of living.

**Fig. 3:**
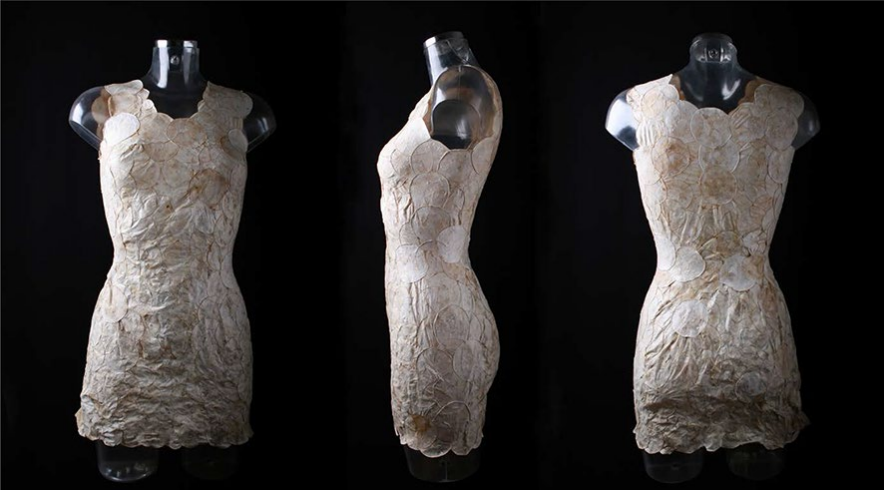
Aniela Hoitink, MycoTEX dress displayed as part of the exhibition FUNGAL FUTURES. © Aniela Hoitink (courtesy of the artist)

Fungi are surprisingly also investigated as new, innovative material in the exhibitions FUNGAL FUTURES – Growing Domestic Bio-Landscape and Possible Tomorrows, where the romantic connection between man and nature acquires new dimensions. Questioning our culture of consumerism, the project *MycoTEX/Mycelium Textile* by artist Aniela Hoitink investigates the use of pure fungal mycelia as dresses (Fig. 3). As opposed to the common consideration of fungi as negative, rather gross entities lurking in dark, damp places, the fungal mycelia in *MycoTEX* are organised in breath-taking, beautiful and decorative meshes. The textile is modular and dynamic and can be rebuilt upon necessity, but is also produced without wastes and is fully compostable. Material is grown and not manufactured in a traditional sense. In a more philosophical discourse, the project *And Who Are You? A Quest for Transparent Living Materials* by Caroline de Roy asks whether fungal structures could replace synthetic materials. By working with a hydrophobin deletion mutant strain of *Schizophyllum commune* (ΔSC3; [9]), which does not produce aerial hyphae, the artist observed the formation of a translucent/transparent mycelium bearing resemblances with the human skin, prompting a reflection about the cycles of growth and decay and the durability of objects—and about our very own identity. In *Continuous Body – The Ephemeral Icon*, Maurizio Montalti exploits the decomposing potential of fungi. The project, subtitled *Infusing life with fungi to trigger a process of final dissolution*, starts from the plastic-degrading property of *Phanerochaete chrysosporium* to criticize the enormity of wastes we produce in the present economic system by increasingly relying on disposable objects. Using a plastic chair as a symbol of this modern culture, the artist documents the catalysis of its dissolution by upholstering it with fungal mycelium, answering the thought-provoking question “…why should not a plastic chair dress up for death?”

**Fig. 4:**
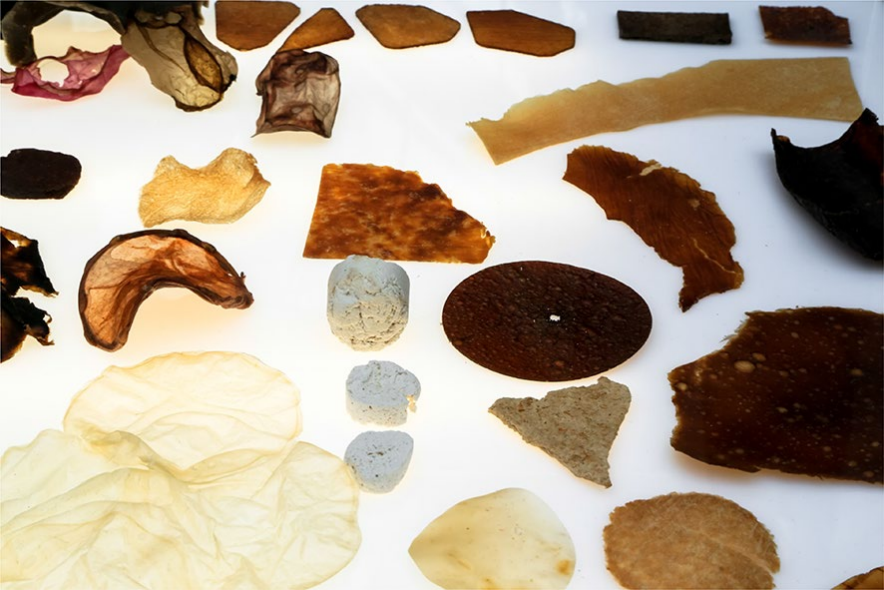
Material samples from the exhibition FUNGAL FUTURES. © Officina Corpuscoli/Maurizio Montalti (courtesy of the artist)

Another key potential of fungi is exploited in *System Synthetics* with the establishment of a synthetic partnership between *P. chrysosporium* and the yeast *Saccharomyces cerevisiae* in an attempt to boost production of bioethanol from plastic wastes. As producers of valuable chemicals while also cleaning up toxic substances, fungi get centre-staged in our modern product-based economy. Indeed, start-up enterprises in the bio-based industry sector like MycoWorks or Mycoplast investigate the commercial production of materials of fungal origin. Characteristics like water repellence, buoyancy, strength and elasticity might lead mycelium-based products to replace leather, clay, wood or some type of plastics (Fig. 4). Even further forward-looking are the works by the artist, designer and writer Alexandra Daisy Ginsberg who reflects on the visions and mind-set of synthetic biologists to use genetic engineering to design new life-forms. In her fictitious patent on a self-inflating antipathogenic membrane pump, featured in the Special Issue “*The Era of Synthetic Biology in Yeast and Filamentous Fungi*” of the journal *Fungal Genetics and Biology* [10], she describes a fungal-inspired synthetic device to detect and treat infections of oak trees caused by *Phytophthora ramorum*. Her fictitious invention hits the nail on the Janus-faced head of both fungi and science. Fungi are our friends and foes and it is up to our imagination and capabilities to exploit or fight them for human welfare. The recent scientific breakthroughs and technologies will likely empower scientists in the near future to decode and reprogram fungi at their will. This, however, requires critical reflection of scientists on their own work and their engagement to discuss the impact of their discoveries on the society and the future of our planet.

## A proper incubator

From these examples, it emerges how fungi are interesting for artists as dynamic, uncontrolled entities that need not to be tamed, but rather investigated and exploited depending on the specific purpose. The mycelium is not collected and displayed, but rather grown and transformed. A transition from the passive display or consumption of fungi as inanimate entities to their active use including the reprogramming of fungal cells as currently done in synthetic biology [11] is present. Contemporary artists, who become inspired by the science of fungi and their potentials in biotechnology, are future-oriented and cultivate a transdisciplinary approach. Themes highly relevant for our modern society such as sustainable living, new materials and ethical considerations are recurrent among the different works. Many of these ventures rise from the collaboration with scientific institutes and individual scientists, who likely enjoy the respect and appreciation of their work by artists, their fresh and unusual perspective on it and because the arts are respected as catalysts in the perception and acceptance of technological and scientific innovations by the society.

When considered in a broader context, the interaction of science and the arts needs a proper environment to let oneself be influenced and to cross-fertilise each other. First of all, a physical place, i.e. a laboratory supplied with the proper equipment in which scientific results and art pieces can be produced is necessary. As academic freedom is as important as artistic freedom and both are international and borderless, no limitations should be put on their practices. When there is the wish to collaborate, scientists and artists can be brought together by various means. To name but a few, the Swiss *Artist in Lab* program for example supports artists who wish to get acquainted with scientific methods and technologies and incorporate them in new projects with long-term residencies in a research institute; the Art Laboratory Berlin invites scientists to regularly teach artists and the interested public on how to cultivate or even manipulate microorganisms in a do-it-yourself (DIY) approach; the *Scientific Delirium Madness* of the Djerassi Resident Artist Program offers a one-month retreat in the San Francisco Bay Area to scientists and artists experienced in working in the other discipline to foster new exchanges and ideas.

To understand the drive of the artist and unmask additional possible similarities between scientific and artistic processes, we talked with Maurizio Montalti, founder of the *Officina Corpuscoli*, a laboratory that investigates the relationships between science, art, society, nature, culture and industry. Interestingly, further intersections among the practice of both disciplines emerged. Exposing his very personal journey, the artist explained how by working on his project *Continuous Body*, where he explored the decomposition and transformation of inanimate and animate matter and questioned the human rituals of death, he stumbled upon the idea of including fungi as a central element of his work. “My purpose changed over time, but both my personal interest in fungi and the importance of observation were central throughout the process. I was fascinated on the spot,” he told us. Starting from a DIY approach, he first experimented with fungi he collected himself or bought from a strain collection in his own bathroom. Realising the technical limitations of his skills and equipment, he sought to collaborate with a fungal research laboratory. “Of the 50 or so researchers that I contacted, only a handful answered. In some cases, I was told that they were too busy doing important stuffs, implicating that my work wasn’t.” Montalti ascribes these reactions to the fact that he uses a different approach than a scientist, and that his questions and purposes are more encompassing than those addressed in a scientific project—what, from a scientist perspective, might be seen as less specific and thus fuzzier. Finally, he successfully started collaborating with Professor Han Wösten from the University of Utrecht—a collaboration that is now nine years in the making—with whom he initiated, curated and produced the exhibition FUNGAL FUTURES. “Even today,” Montalti adds, “the process is more important than the results themselves. And yet, what motivates me is to be able to make an impact rather than to secure a possible financial return.” These motivations and the necessity to search for success in failure reverberate with the everyday life of a research laboratory and the long-term goal of any impactful scientific project.

## The end

“Since we’re all going to die, it’s obvious that when and how don’t matter,” Albert Camus wrote in *The Stranger* (1942). This statement is radically contradicted by the artist and inventor Jae Rhim Lee who cares about the “how.” With the assumption that our bodies are enriched in toxins and that funeral practices severely harm the environment, she designed and developed a burial suit infused with fungal spores in her own DIY lab to ensure that both the body and the toxins within become fully decomposed after her death. “I started collecting my hair, nails and skin so I could pick the best mushrooms to become infinity mushrooms, to recognise and eat my body after I die,” she stated in an interview with *New Scientist* [12] after presenting her vision at the TEDGlobal conference in 2011 [13]. A must-see video! Enthralled by this idea, she studied mycoremediation, collaborated with experts from science, arts, design and funeral industries, started the Infinity Burial Project and founded the burial company Coieo in 2014 (www.coeio.com). The first adopter, Dennis White, expressed his interest to become buried with the “Infinity Burial Suit” in 2015 because he was diagnosed with a terminal illness and wanted to become buried in an environmentally-friendly way. “I want to go out with a bang, like I’ve lived most of my life,” he said in a documentary about the suit. He died recently, in September 2016, and became the first-ever person to be buried in a “Infinity Burial Suit” without embalming or even a coffin. With this end of his life he might pave the way for success of a visionary and astonishing fungal product inspired by an artist.
